# Early Alterations in De Novo Parkinson’s Disease Revealed by Diffusion Tensor Imaging: Preliminary Study

**DOI:** 10.3390/diagnostics15070841

**Published:** 2025-03-25

**Authors:** Francesca Di Giuliano, Noemi Pucci, Maria Lina Serio, Eliseo Picchi, Silvia Minosse, Valentina Ferrazzoli, Valerio Da Ros, Tommaso Schirinzi, Matteo Conti, Roberta Bovenzi, Davide Mascioli, Francesco Garaci

**Affiliations:** 1 Neuroradiology and Diagnostic Imaging Unit, Department of Biomedicine and Prevention, University of Rome Tor Vergata, Via Montpellier 1, 00133 Rome, Italy; noemi.pucci@alumni.uniroma2.eu (N.P.); marialinaserio@gmail.com (M.L.S.); eliseo.picchi@uniroma2.eu (E.P.); silvia.minosse2@gmail.com (S.M.); valentinaferrazzoli@hotmail.it (V.F.); valeriodaros@hotmail.com (V.D.R.);; 2 Unit of Neurology, Department of Systems Medicine, Tor Vergata University of Rome, 00133 Rome, Italy; t.schirinzi@yahoo.com (T.S.); matteoconti92@gmail.com (M.C.); roberta.bovenzi@gmail.com (R.B.); davidemascioli@outlook.it (D.M.); 3 San Raffaele Cassino, Via Gaetano di Biasio, 1, 03043 Cassino, Italy

**Keywords:** Parkinson’s disease, de novo Parkinson’s disease, diffusion tensor imaging, fractional anisotropy, thalamus and putamen

## Abstract

**Background/Objectives**: Parkinson’s disease (PD) is characterized by progressive neurodegeneration affecting both motor and non-motor functions. Identifying early alterations in PD patients before the onset of dopaminergic therapy is crucial for understanding disease progression and developing targeted interventions. This study aimed to investigate early changes in the putamen and thalamus in de novo PD patients using diffusion tensor imaging (DTI) compared to healthy controls. **Methods**: Thirty-one de novo PD patients and thirty-three healthy controls underwent DTI scanning. Tract-based spatial statistics were used to compare fractional anisotropy (FA) values between groups. **Results**: De novo PD patients exhibited significantly lower FA values in the right thalamus compared to controls, suggesting alterations in neuronal integrity or fiber degeneration in the early stages of the disease. However, no significant differences were demonstrated for FA values in the putamen between groups. **Conclusions**: We demonstrated that the FA value in the right thalamus was lower in PD compared with healthy controls. These findings highlight the potential of DTI as a non-invasive tool for detecting early neural changes in PD patients. Further studies would be helpful to assess the clinical utility of serial FA measurements of the subcortical gray matter in objective quantification of disease progression and monitoring of the therapeutic response.

## 1. Introduction

Parkinson’s disease (PD) is a common neurodegenerative disorder, currently affecting 1% of the population over the age of 60 [1]. PD is a common neurodegenerative disorder, whose neuropathological hallmarks are the loss of dopaminergic nigral cells and the brain accumulation of α-synuclein (α-syn) positive Lewy bodies. PD is a heterogeneous disorder, either in terms of pathogenic mechanisms or clinical presentation. Indeed, several motor and non-motor disturbances may affect patients along the disease course. Despite such dramatic heterogeneity, the motor syndrome (bradykinesia, rigidity, and tremor) still remains the core feature of PD, which allows for the disease [2,3].

The pathophysiology of motor symptoms in PD, as well as other non-motor symptoms, is largely dependent on degeneration of the dopaminergic pathway and disruption of basal ganglia circuits, driven by neurotransmitter imbalance and progressive neurodegeneration [2]. PD is characterized by the presence of Lewy bodies and neurites in both neuronal bodies and axons, including aggregates of the α-synuclein protein, which has been linked to axonal changes and subsequent neuronal degeneration [4].

The theory that retrograde axonal degeneration may be the underlying mechanism of PD has gained considerable support in recent years. This hypothesis posits that the accumulation of α-synuclein may have its genesis in presynaptic terminals. This, it is suggested, gives rise to modifications in axonal transporters and subsequent axonal degeneration, before affecting neurons [5,6]. The heterogeneous nature of PD implies the existence of widespread alterations in brain structure, and the involvement of axonal components is regarded as a pivotal factor in PD’s pathological mechanisms.

Some studies have indicated that the structural integrity of the white matter (WM) is compromised prior to the observable reduction in cortical thickness [7,8].

Accordingly, a deeper examination of structural changes within the subcortical structures of the brain might enhance the understanding of the disease and also support the identification of novel targets useful for biomarkers and therapeutic development.

It is crucial to study patients with PD in the early stages of the disease to assess the subtle and early alterations that occur in brain structure. The present study is thus centered on patients with de novo Parkinson’s disease who have been newly diagnosed, with a disease duration of less than 24 months and no prior history of therapy involving dopaminergic drugs.

Conventional MRI makes it very difficult to distinguish between Parkinson’s patients and healthy controls. However, advances in MRI acquisition techniques have proven to be valuable non-invasive imaging tools for aiding in diagnosis, prognosis, and monitoring disease progression and/or treatment [9,10].

Diffusion Tensor Imaging (DTI) is the most commonly utilized technique for the extraction of microstructural features, such as fractional anisotropy (FA) [11], which permits the in vivo analysis of microstructural alterations in axonal involvement. DTI is one of the most utilized metrics in the investigation of Parkinson’s disease patients [12]. FA is a metric that specifically measures the directionality of random water diffusion, axonal integrity, and the degree of axonal myelination. Several DTI studies have documented microstructural alterations in both the basal ganglia and the mesencephalic regions [12].

The majority of studies have focused on analyzing Parkinson’s patients undergoing antiparkinsonian drug therapy. Conversely, only a limited number of studies have evaluated de novo PD patients using DTI technique on T3 MRI, which enhances the ability to detect microstructural changes in the brain [12].

In recent years, DTI protocols have been employed in PD to track pathological changes in the brain, thereby providing a valuable source of biomarkers. Nevertheless, the considerable variability in results across the literature, attributable to differences in methodology or patient characteristics, underscores the need for further investigation in this field.

In particular, the early disease stages are those deserving the greatest effort, as they represent an ideal time-window in which disease-modifying interventions may be more effective [13,14]. Accordingly, we performed a DTI study on a de novo PD population (newly diagnosed, untreated patients), with the objective of examining structural integrity within the thalami and the putaminal nuclei, which represent the main critical stations of the basal ganglia circuits [15]. We hypothesized that altered microstructural integrity exists within the thalami and the putaminal nuclei due to complex basal ganglia-thalamocortical circuitry neurodegeneration. The aim of this investigation was to identify early changes using in vivo neuroimaging study that could prove useful for biomarker purposes, with the potential to act as biomarkers in clinical trials, since treatment strategies are different between the early and late stages of PD [16].

## 2. Material and Methods

### 2.1. Subjects

The study included a total of 61 subjects comprising 30 de novo PD patients and 31 healthy control subjects, recruited at the Parkinson Centre, Department of Systems Medicine, of the University Hospital of Rome “Tor Vergata”.

We recruited patients diagnosed with idiopathic PD, according to 2015 Movement Disorder Society (MDS) diagnostic criteria [17]. Patients were eligible for the current study if they fulfilled the following inclusion criteria: (i) disease duration < 24 months; (ii) no history of taking therapy with dopaminergic drugs (iMAO, L-Dopa, Dopamine agonists, iCOMT, anticholinergic agents, amantadine); (iii) no history of epilepsy or other conditions (i.e., brain tumors, stroke, infections, etc.); (iv) no cognitive impairment, as defined by a Mini-Mental State Examination (MMSE) score above 25 [18]; (v) morphological MRI without brain parenchymal lesions; and (vi) no other neurological diseases except PD. De novo PD patients included 22 men and 9 women, with an average age and a standard deviation age of 60.0 ± 11.4, respectively. Each de novo PD patient was evaluated using the MDS-Unified Parkinson’s Disease Rating Scale Parts 3 (MDS-UPDRS III) and the Hoehn and Yahr (H&Y) scales to assess motor symptoms, the Mini-Mental State Examination (MMSE) and Montreal Cognitive Assessment (MoCA), adjusted for age and educational level, to evaluate cognitive deficits, and the Non-Motor Symptoms Scale (NMSS) to assess non-motor symptoms. Healthy controls were age-matched subjects with normal brain MRI and no previous history of neurological and neuropsychiatric disorders. For all subjects, we collected medical history, demographics and anthropometric data (weight in kg, height in cm, body mass index (BMI)).

Healthy control subjects included 16 men and 17 women, with an average age and a standard deviation age of 56.6 ± 13.8, respectively. No statistically significant differences were found in age (*p* = 0.48, Mann–Whitney U-test) and gender (*p* = 0.07, Chi-square test) between the two groups. Table 1 summarizes the main clinical and demographic features of the study population. No significant differences resulted for anthropometrics between de novo PD patients and healthy control subjects. In de novo PD group, the prevalence of males was significantly higher (*p* = 0.02).

The study was conducted in accordance with the Declaration of Helsinki, and approved by local institutional ethics committee: Comitato Etico Indipendente Fondazione PTV Policlinico Tor Vergata (D.M. 8 febbraio 2013). Ethical code and approval date: Progetto MNESYS “MUR PE0000000 Spoke6 WP4” R.S 133.23 (6 June 2023).

### 2.2. MRI Images Acquisition

The maximum time interval between MRI and clinical assessment examinations was one week.

All subjects underwent brain MRI imaging on a 3T superconducting magnet (Achieva 3T Intera, Philips Healthcare, Best, The Netherlands) with a 32-channel sensitivity-encoding head coil.

High-resolution T1-weighted anatomical images were acquired using a magnetization-prepared rapid gradient echo (MPRAGE) 3D sequence with the following acquisition parameters: voxel size = 1 × 1 × 1 mm^3^, repetition time (TR) = 6.5 ms, echo time (TE) = 2.9 ms, flip angle = 9°, and field of view (FOV) = 256 × 256 mm^2^.

DTI images were acquired using a single-shot spin-echo echo-planar imaging (SE-EPI) sequence with the following acquisition parameters: FOV = 228 × 228 mm^2^, matrix = 116 × 112 voxels, TE = 93 ms, TR = 8792 ms, slice thickness = 2 mm, 66 slices, no gap, SENSE reduction factor R = 1.3. A b-value of 1000 s/mm^2^ was applied, acquired along 16 non-coplanar and non-collinear directions for diffusion encoding (8 for each b-value different from 0), in addition to a set of images with b = 0 s/mm^2^.

Additionally, we acquired Axial T2-weighted Turbo Spin Echo images (TR = 3000 ms, TE = 80 ms, and thickness/gap = 4/1 mm), Fluid Attenuation Inversion Recovery (FLAIR) 3D (voxel size = 1.12 × 1.12 × 1.12 mm^3^, TR = 4800 ms, TE = 340 ms, flip angle = 40°, FOV = 250 × 250 mm^2^) and axial Fast Field Echo (FFE) (TR = 904 ms, TE = 16.11 ms, thickness/gap = 4/1 mm), which were employed by an expert neuroradiologist (FG) to exclude the presence of pathological findings.

### 2.3. DTI Analysis

**Figure 1:**
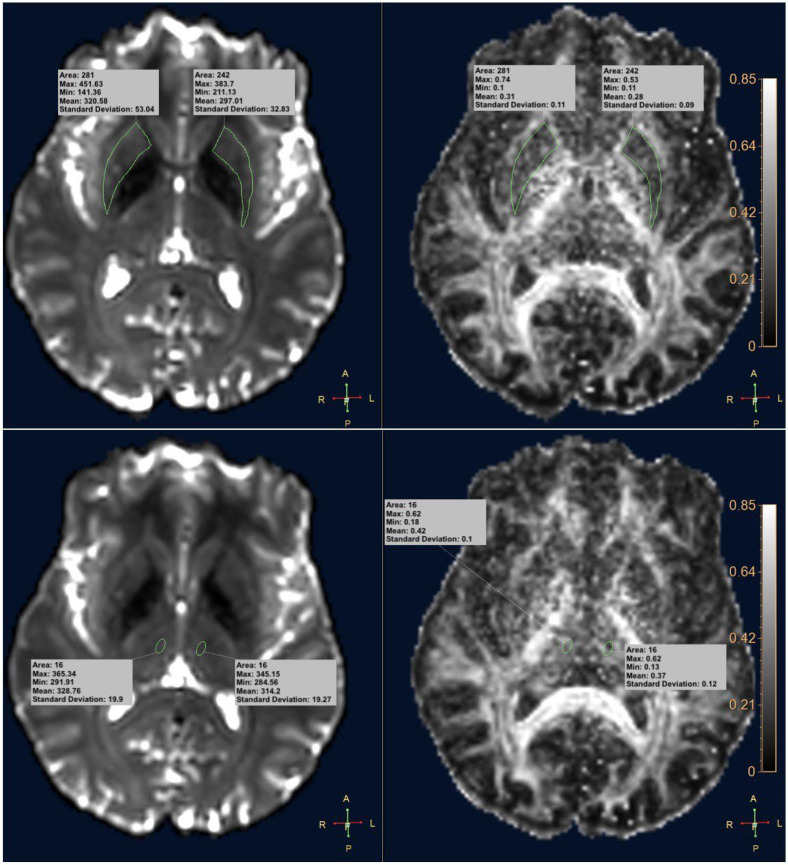
Interface of the MR Diffusivity tool of the IntelliSpace V 9.0 software used for the analysis of diffusion images. By selecting the values of b = 0 s/mm^2^ and b = 1000 s/mm^2^, the FA maps co-registered to the diffusion-weighted images were generated. Freehand ROIs were drawn on the putamen and elliptical ROIs on the thalami on the b = 0 s/mm^2^ images. For each ROI, the FA values obtained were maximum, minimum, mean and standard deviation. Unit of measurement area mm^2^.

The DTI study analysis was performed using the advanced “MR Diffusivity” application of IntelliSpace (ISP) Portal Version 9.0 software (Philips Healthcare, Best, The Netherlands), which generated FA maps using values b = 0 s/mm^2^ and b = 1000 s/mm^2^. Regions of interest (ROIs) were drawn on the anatomical structures under study on the b = 0 s/mm^2^ map co-registered with the FA map. ROIs on the putamen were traced freehand along the profile of the putaminal nucleus on each cerebral hemisphere, right and left, with respective average area/standard deviation values of 193 mm^2^/40.97 on the right and 187.91 mm^2^/46.28 on the left. Elliptical ROIs were drawn on the thalamus positioned in the medio-dorsal region on each cerebral hemisphere, with average area/standard deviation values of 15.81 mm^2^/0.35. For each ROI, the software computed four FA values: maximum, minimum, mean, and standard deviation (Figure 1).

### 2.4. Statistical Analysis

Statistical analysis was performed using the Statistical Package for the Social Sciences (SPSS) for Windows, version 15.0 (SPSS, Chicago, IL, USA). Descriptive statistics included mean ± standard deviation for parameters with normal distributions (confirmed by histograms and Kolgomorov–Smirnov test) and median with range (min.–max.) for variables with non-normal distributions. Sample comparisons were conducted using one-way analysis of variance (ANOVA). The agreements between the FA estimations obtained with de novo PD patients and healthy controls, for right and left thalamus, were assessed by Bland–Altman plots. Differences between the parameter values were plotted against their averages, the horizontal line representing the mean difference and the dotted horizontal lines representing the upper and lower 95% limits (95% confidence intervals [Cis]) of agreement. Spearman’s rank correlation analysis was performed to evaluate the association between FA values and clinical variables, adjusted for age and sex. In order to exclude false positive results that may arise from multiple testing, a false discovery rate (FDR, alpha = 0.05) procedure was applied. A *p*-value < 0.05 (FDR corrected) was considered statistically significant.

## 3. Results

**Figure 2:**
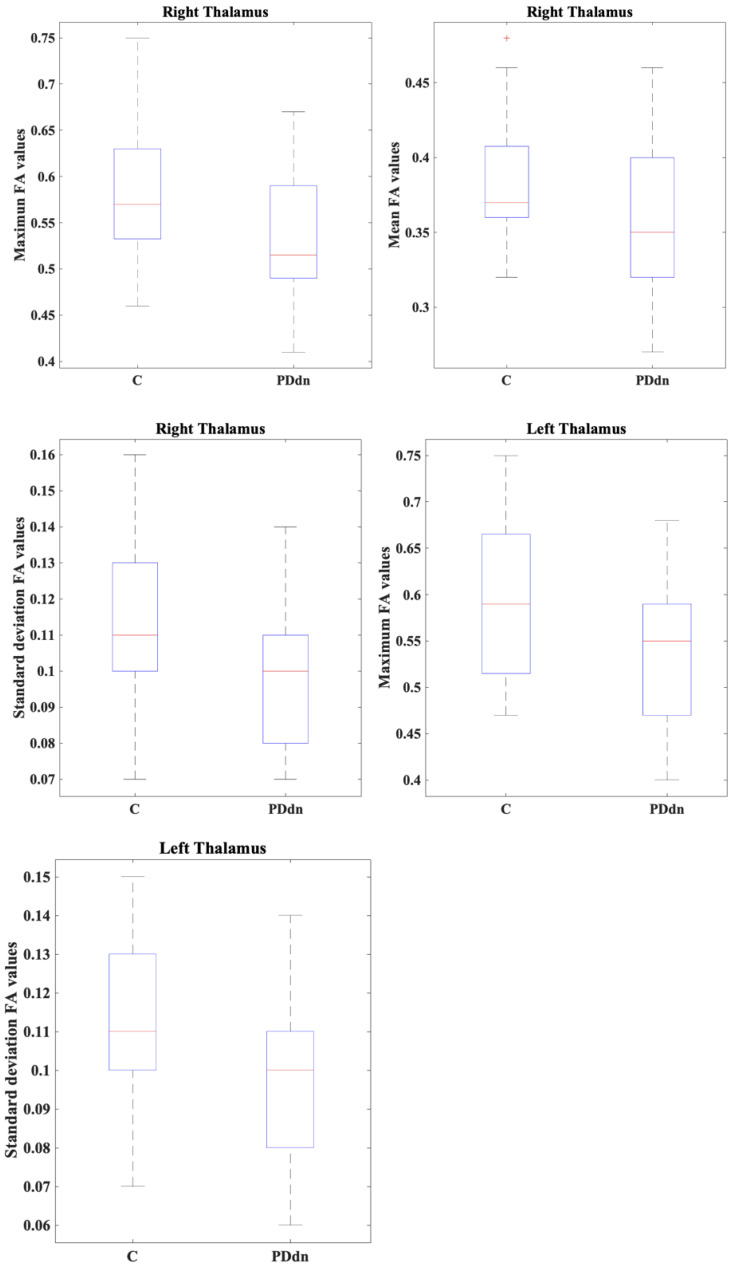
Box-and-whisker plots of FA values in the right and left thalamus between de novo PD patients (PDdn) and healthy controls (C).

Summary statistics of FA values for right and left putamen and thalamus in the two subject groups (de novo PD patients and healthy control subjects) are reported in Table 2. De novo PD patients showed significantly lower FA values (maximum, mean and standard deviation) in the right thalamus, compared to healthy control subjects (*p* = 0.018, *p* = 0.045 and *p* = 0.010, respectively), while they exhibited lower FA values (minimum) without reaching significancy (*p* = 0.640). De novo PD patients showed significantly lower FA values (maximum and standard deviation) in the left thalamus, compared to healthy control subjects (*p* = 0.012 and *p* = 0.010, respectively), while they exhibited lower FA values (mean) without reaching significancy (*p* = 0.387) and higher FA values (minimum) without reaching significancy (*p* = 0.295). The FA values in right and left putamen were not significantly different between the novo PD patients and healthy controls for all extracted metrics (maximum, minimum, mean, and standard deviation). Statistically significant FA values for the right and left thalamus in the two subject groups (de novo PD patients and healthy control subjects) are illustrated in Figure 2.

Spearman’s rho and *p*-values summary of correlation analysis were statistically significant without FDR correction in the right and left putamen, as shown in Table 3. After FDR correction, we found no statistically significant associations between FA metrics and clinical parameters.

## 4. Discussion

The present study examined DTI parameters in the thalamic and putamen nuclei within a population diagnosed with de novo Parkinson’s disease. DTI is commonly used to identify changes in white matter [19], but it can also detect abnormalities in gray matter. It may be able to detect these changes earlier than traditional MRI methods. Studies using DTI have shown changes in diffusivity in subcortical structures and decreased FA in the substantia nigra in individuals with Parkinson’s disease [20,21,22]. It has been shown that neurodegeneration, in the form of deficits in subcortical and cortical gray matter tissue, occurs at greater levels at advanced disease stages [23,24]. The objective of the study was to identify early changes in brain structure that could facilitate a more profound comprehension of the pathophysiology of Parkinson’s disease. Furthermore, the detection of specific microstructural abnormalities in brain regions has the potential to serve as biomarkers that could aid in the early diagnosis of the disease and in the monitoring of its progression.

It is noteworthy that the FA values in the examined thalamic regions were significantly reduced in de novo PD patients compared to age-matched healthy controls. The reduction in FA in the thalamus of de novo PD patients may indicate the altered integrity of neuronal cells or a potential degeneration of fibers in the bilateral mediodorsal thalamus in the early stages of the disease compared to healthy controls. This finding may be indicative of microstructural alterations in white matter fibers associated with the mediodorsal thalamus, including both those projecting to and those originating from it. However, higher FA and lower mean diffusivity (MD) have recently been observed throughout the white matter skeleton, including the posterior thalamic radiation, in the early stages of PD, consistent with the early compensatory changes associated with the disease [19].

The thalamus is a critical station in motor networks. Indeed, the striato-thalamo-cortical (STC) circuitry is significantly affected by dopamine deficiency in PD, leading to a dysfunctional signaling that contributes to pathophysiology of motor disturbances. Furthermore, pathological studies have demonstrated that the size and shape of the thalamic subnuclei undergo substantial alterations in PD [15]. Consequently, the DTI alterations in the thalamus observed in this study accurately reflect these morpho-functional modifications that occur in the early stages of the disease.

Previous authors similarly observed a decreased FA in thalamus in PD patients [20,25,26]. In a study by Zhang and colleagues [20], it was found that PD is associated with a higher rate of FA reduction compared to that observed in normal aging. This reduction was primarily observed in the substantia nigra, the thalamus, and midbrain regions. Additionally, it was reported that DTI alterations in the substantia nigra of patients with PD correlated with dopamine deficiency and reduced α-synuclein levels in the CSF.

A voxel-based analysis conducted by Li et al. [25] revealed a reduction in FA values in the bilateral mediodorsal thalamus in patients with PD and depression compared to those without depressive symptoms. This finding suggests a potential correlation between FA in the mediodorsal thalamus and the presence of depressive symptoms in these patients. The mediodorsal thalamus receives dopaminergic projections, which are crucial for modulating thalamic activity. Additionally, dopamine loss has been observed in the limbic system of PD patients with depression compared to PD patients without depressive symptoms, including in the mediodorsal thalamus [27].

However, our results contradict some other studies that have not shown a significant reduction in FA values in the thalamus between PD patients and healthy controls. The majority of these studies examined subjects with advanced PD who were undergoing pharmacological treatment. This represents a significant confounding factor, given the lack of certainty as to whether these changes can be attributed to the pathology itself or the therapeutic intervention. For instance, Oliveira et al. [28] observed that PD patients undergoing therapy exhibited markedly diminished FA values in the substantia nigra when compared to healthy controls. However, no significant reduction in FA was observed in the thalamus.

This difference may suggest a reorganization of circuits involving the thalamus, following exposure to antiparkinsonian therapies. The utilization of levodopa has been demonstrated to instigate a multifaceted sequence of electrophysiological and molecular phenomena, which in turn precipitate aberrant plasticity within the cortico-basal ganglia system. This process has been identified as a pivotal contributor to the pathophysiology of Levodopa-induced dyskinesias LIDs [29].

The study conducted by Planetta and colleagues [30] was the first to examine the integrity of thalamic nuclei projections in de novo PD using in vivo DTI tractography. In alignment with our findings, they observed a reduction in FA values in the fibers projecting from the anterior nucleus, dorsomedial nucleus, and ventral anterior nucleus of the thalami.

A substantial number of studies have reported heterogeneous findings in relation to DTI changes. These studies have included a wide range of participants in terms of age, disease severity, medication status and the presence of non-motor symptoms. It can be hypothesized that the clinical heterogeneity of the study population is reflected in the inconsistent findings of DTI abnormality across previous studies [12].

Furthermore, although other authors employed a comparable data analysis approach, utilizing manual ROI placement, the majority of previous studies were conducted on 1.5T scanners. Consequently, the different observed outcomes can be attributed to the enhanced sensitivity of the scanner, which is a consequence of the higher magnetic field intensity. In addition, scanning parameters, including the number of encoding directions and the maximum b-value, have been demonstrated to exert a substantial influence on DTI measurements.

In our study, DTI parameters did not differ between patients with de novo Parkinson’s disease and healthy controls in the putamina. Indeed, pathological changes in the putamina, as trackable by MRI techniques, might be more appreciable in later disease stages rather than at disease onset, as suggested in previous studies [21,31]. Surova et al. found a selective reduction in FA in the putamen in PD over a two-year period that correlated with increasing daily L-dopa equivalent dose at baseline and follow-up [32]. Although Surova et al. used the DKI technique, these results agree with ours, as we found no differences in FA values in the putamina between PD patients who never received therapy and healthy controls. Thus, although it is not an early marker of the disease, it may be that the decreased FA in the putamen at follow-up has some clinical relevance and it may represent a marker of disease progression. However, long-term follow-up studies in PD using DTI are limited due to the inherent clinical challenges of recruiting elderly patients, the nature of this chronic debilitating disease in its later stages, and MR scanner upgrades over time [33].

Regarding the correlation analysis with clinical variables, no significant findings resulted because of either the sample size or the specific clinical features of the population (homogeneous early-stage group with mild clinical impairment).

This study has several limitations, such as the small sample size and the gender imbalance in the subject sample compared to the control group. FA values in DTI analysis were obtained manually, which may have introduced measurement bias. We adopted a simple and practical approach to draw the ROIs, which were drawn freehand, potentially affecting reproducibility and introducing measurement bias. Reproducibility assessment was not possible as the analyses were conducted by a single radiologist. Further studies utilizing larger samples and longitudinal follow-ups are required to evaluate the evolution of microstructural alterations in patients with PD and the effects of treatment on brain network organization.

## 5. Conclusions

The observed alterations in the DTI metrics in the subcortical regions may be considered an early indicator of the pathophysiology of de novo PD in patients with no history of exposure to antidopaminergic therapies. These alterations may prove useful for understanding potential neural mechanisms and characterizing subtypes of PD patients in relation to cognitive dysfunctions or other non-motor symptoms. The identification of these early alterations in de novo PD patients before the onset of dopaminergic therapy could be crucial for understanding disease progression and developing targeted interventions. The DTI technique, which is widely accessible and non-invasive, could serve as an additional tool for studying early PD in clinical trials, including the evaluation of new therapies.

## Figures and Tables

**Table 1:** Clinical and demographic characteristics of the study population (de novo PD patients and healthy control subjects). The data are expressed with mean and standard deviation values. Age is expressed in years and the duration of the disease in months.

	De Novo PD (*n* = 31)	Control Subjects (*n* = 33)
Male/Female	22/9	16/17
Age	60.0 ± 11.4	56.6 ± 13.8
BMI	25.4 ± 3.0	24.2 ± 3.0
Disease duration (months)	18.0 ± 9.9	-
H&Y	1.8 ± 0.6	-
MDS-UPDRS III	27.1 ± 11.1	-
MoCA	25.4 ± 3.4	-
MMSE	27.9 ± 2.0	-
NMSS	32.1 ± 26.6	-

Abbreviations: BMI, body mass index; H&Y; Hoehn and Yahr scales; MDS-UPDRS III, MDS-Unified Parkinson’s Disease Rating Scale Parts 3; MoCA, Montreal Cognitive Assessment; MMSE, Mini-Mental State Examination; NMSS, Non-Motor Symptoms Scale.

**Table 2:** Comparison of FA values between de novo PD patients and healthy controls. The results are expressed with mean and standard deviation values.

Region		De Novo PD Patients	Control Subjects	*p*
Right Putamen	Max	0.711 ± 0.079	0.710 ± 0.067	0.985
	Min	0.117 ± 0.038	0.113 ± 0.025	0.652
	Mean	0.366 ± 0.036	0.364 ± 0.033	0.839
	Std	0.121 ± 0.014	0.126 ± 0.012	0.185
Left Putamen	Max	0.681 ± 0.084	0.689 ± 0.070	0.672
	Min	0.115 ± 0.034	0.117 ± 0.033	0.868
	Mean	0.360 ± 0.035	0.362 ± 0.032	0.764
	Std	0.119 ± 0.013	0.011 ± 0.08	0.989
Right Thalamus	Max	0.541 ± 0.077	0.586 ± 0.075	0.018 *
	Min	0.196 ± 0.061	0.213 ± 0.184	0.640
	Mean	0.359 ± 0.052	0.364 ± 0.033	0.045 *
	Std	0.098 ± 0.024	0.114 ± 0.021	0.010 *
Left Thalamus	Max	0.541 ± 0.077	0.595 ± 0.085	0.012 *
	Min	0.207 ± 0.055	0.193 ± 0.049	0.295
	Mean	0.366 ± 0.040	0.374 ± 0.033	0.387
	Std	0.098 ± 0.024	0.114 ± 0.021	0.010 *

Abbreviations: Max, maximum; Min, minimum; Std, standard deviation. * *p* < 0.05.

**Table 3:** Correlation coefficients between FA values and clinical variables with *p* value < 0.05 without FDR correction. The correlation between this variable disappeared after FDR correction.

Regions	FA Measures	Clinical Variables	Rho	*p* Value
Left putamen	Mean FA	MMSE	0.43	0.039
Right putamen	Mean FA	H&Y	−0.52	0.007
Left putamen	Mean FA	MDS-UPDRS III	−0.42	0.038
Right putamen	Std FA	NMSS	−0.41	0.049

Abbreviations: H&Y; Hoehn and Yahr scales; MDS-UPDRS III, MDS-Unified Parkinson’s Disease Rating Scale Parts 3; MMSE; Mini-Mental State Examination; NMSS, Non-Motor Symptoms Scale; Std, standard deviations.

## Data Availability

The original contributions presented in this study are included in the article. Further inquiries can be directed to the corresponding author.
